# Translating the multifaceted use of liquid biopsy to management of early disease in pancreatic adenocarcinoma

**DOI:** 10.3389/fonc.2025.1520717

**Published:** 2025-03-13

**Authors:** Madison Cox, Dominic Vitello, Akhil Chawla

**Affiliations:** ^1^ Division of Surgical Oncology, Department of Surgery, Northwestern University Feinberg School of Medicine, Chicago, IL, United States; ^2^ Northwestern Medicine Cancer Centers, Northwestern Medicine Regional Medical Group, Winfield, IL, United States; ^3^ Northwestern Quality Improvement, Research and Education in Surgery, Department of Surgery, Northwestern University Feinberg School of Medicine, Chicago, IL, United States; ^4^ Robert H. Lurie Comprehensive Cancer Center of Northwestern University, Chicago, IL, United States

**Keywords:** pancreatic adenocarcinoma, liquid biopsy, circulating tumor DNA, digital droplet PCR, next generation sequencing, mutant KRAS, minimal residual disease, targeted therapy

## Abstract

Pancreatic ductal adenocarcinoma (PDAC) is a leading cause of cancer-related mortality, primarily due to late stage at diagnosis. This review examines the multifaceted applications of liquid biopsy and circulating tumor DNA (ctDNA) analysis in the diagnosis and management of PDAC. We review the current literature on the technological advancements in liquid biopsy analysis such as next generation sequencing (NGS) and digital droplet PCR (ddPCR) as well as multi-omics technologies, highlighting their potential for accurate molecular subtyping through ctDNA analysis. This review highlights the significant role of ctDNA in the assessment of tumor behavior, disease subtyping, prediction and monitoring of treatment response, and evaluation of minimal residual disease. We discuss the implications of integrating liquid biopsy techniques into clinical practice as well as its challenges and limitations. By drawing insights from recent studies, this review aims to provide a comprehensive overview of how liquid biopsy and ctDNA analysis can enhance early disease management strategies in PDAC. We underscore the need for additional prospective studies and clinical trials to validate its feasibility and accuracy in order to establish clinical utility, with the ultimate goal of routine incorporation into practice to improve patient outcomes and transform the treatment landscape for PDAC.

## Introduction

In recent years, liquid biopsy has moved to the forefront of biomarker research aiming to revolutionize the treatment of many solid cancers (1). With its unique advantages over traditional tissue biopsy in providing earlier diagnosis, personalized treatment, better affordability, and more, liquid biopsy certainly holds promise in future clinical practice for the management of many patients with cancer. This is particularly true for patients with pancreatic ductal adenocarcinoma (PDAC), for which progress has been slower than in other malignancies. Liquid biopsy offers a non-invasive alternative to tissue biopsy that captures and utilizes genetic information for diagnosis that can be obtained from multiple body fluids, including blood, urine, saliva, ascites, pleural fluid, cerebrospinal fluid (2–4). In this review, however, the focus will be predominantly on blood-based liquid biopsy. Evaluation of genetic information leading to early cancer diagnosis and management involves analysis of cell-free DNA analyzed via liquid biopsy through various laboratory methods. Cell-free DNA (cfDNA) refers to DNA fragments found freely in body fluids that can be derived from both healthy and cancerous cells. Fragments of cfDNA derived from healthy cells average around 160 base pairs in length, and are distinguishable from tumor-associated cfDNA, termed circulating tumor DNA (ctDNA), which averages a slightly shorter fragment length of 140 base pairs (5, 6).

The consensus on the origin of ctDNA involves primary tumor cells that have undergone apoptosis, necrosis, or are actively metastasizing (6). In addition to ctDNA, circulating tumor cells (CTCs) as well as other cellular components such as RNA or proteins may be present and provide information indicative of malignancy and of the primary tumor’s characteristics (7). With a half-life of less than 2 hours, ctDNA analysis provides an accurate reflection of the current genetic landscape and behavior of the primary tumor in real-time (8). From first identification of its relevance in cancer patients in the landmark paper by Leon et al. in 1977, ctDNA has grown in its significance to be at current the most established liquid biopsy analyte for use in cancer management with multiple large clinical trials and FDA-approved device panels evaluating its validity in early diagnosis, prognostication, targeted treatment selection, and residual disease monitoring (9, 10).

## Pancreatic ductal adenocarcinoma: background and current challenges

Among solid malignancies, PDAC is highly aggressive. Currently for patients who are diagnosed with PDAC, the five-year overall survival rate is nearly 13% (11). Margin-negative surgical resection provides the best possibility of long-term control and possible cure of disease. However, only 20-30% of PDAC cases are considered resectable at diagnosis, yet still face a five-year survival rate of less than 50% due to high rates of recurrence (12). Despite recent efforts in optimizing diagnostic and surgical techniques, there have been minimal improvements in survival. With the prevalence of PDAC continuing to rise, developing methods for earlier diagnosis and optimal treatment are vital to improving outcomes for those diagnosed.

Early diagnosis of PDAC is difficult for many reasons. For one, screening of individuals at average risk for pancreatic cancer is not standard of care. Around 70-90% of individuals who will be diagnosed with PDAC are at average risk (13). Many of the most common factors are only weakly associated with an increase in risk and are largely related to a patient’s lifestyle (e.g. smoking use, uncontrolled diabetes, alcohol use, obesity). Screening for PDAC often involves time-consuming and expensive procedures such as yearly MRI, esophagogastroduodenoscopy and endoscopic ultrasound, repeat fine needle aspirations and blood draws that have not been shown to identify enough early PDAC cases to justify the extent of invasive procedures that individuals will undergo throughout their lifetime (13, 14). The United States Preventive Services Task Force has found minimal benefit to screening asymptomatic individuals of average risk given lack of informative screening methodologies and poor prognosis even when caught at an early stage, and therefore routine screening is not employed for individuals without defined genetic or hereditary risk (14). Monitoring of pancreatic intraepithelial neoplasms (PanINs), histologically identified areas within the pancreas that in rare cases progress to malignancy, is also not a possibility as these lesions are not directly evaluable except in pathologic specimens, and data is lacking to clearly understand the percentage of patients with PanINs that will progress to PDAC. Some studies have estimated the likelihood of PanIN progression to PDAC to be less than 2% and taking up to 35 years or more, making screening of these lesions minimally beneficial even if feasible (15, 16). Additionally, PanINs are found in nearly three-fourths of pancreatectomy and autopsy specimens by age 80, as well as in multiple individuals in their 20’s with low rates of progression to cancer (17). Conversely, another well-known precursor lesion in PDAC, intraductal papillary mucinous neoplasms (IPMNs), are estimated to be detectable in around 3%–6% of the general population and 10% of the population over 70, and can be monitored as compared to PanINs which cannot. Additionally, IPMNs demonstrate much higher progression rates to malignancy depending on the subtype, with rates as high as 90% in mixed-type types and as low as 3.3% in branch-duct types (18). However, there is difficulty in differentiating IPMNs that will progress to PDAC from those that will not, and further studies are needed to identify genetic differences, histological differences, or both to clearly stratify patients and avoid overtreating when unlikely to mitigate risk.

For the 5-10% of diagnosed PDAC patients that have known genetic risk based on family history (defined as at least one first-degree relative or two second-degree relatives diagnosed with pancreatic cancer in their lifetime) as well as an additional 3-5% of individuals that carry a predisposing genetic mutation (most commonly Hereditary Breast and Ovarian Cancer Syndrome (BRCA1/2), Fanconi Anemia, Familial Adenomatous Polyposis (FAP), Peutz-Jeghers Syndrome, Li-Fraumeni Syndrome, Familial Atypical Multiple Mole Melanoma Syndrome (FAMMM) or Lynch Syndrome (MLH1, MSH2/6 or PMS2 variants)), there is identified benefit from screening (19). The large multi-institutional CAPS5 study employing screening of high-risk populations reported detection of nine PDAC cases, eight of which were found at a resectable stage and exhibited markedly improved overall survival of 9.8 years compared to 1.5 years for patients diagnosed outside of high-risk surveillance (20).

For the large remaining number of average-risk individuals who will go on to develop PDAC, there is a massive need for improved early diagnostic methods where ctDNA analysis provides an attractive solution. In the past two decades, the incidence of early stage PDAC has increased significantly with demonstrated improvements in overall survival with current methodologies, however further improvements in diagnosis at early stages of this disease are necessary to improve its dismal survival rate and improve outcomes for individuals who face this diagnosis.

## Circulating tumor DNA: a non-invasive tool for early PDAC detection

Diagnosis of PDAC often involves identification of a pancreatic mass on radiographic imaging with the subsequent need for tissue biopsy. However, as described previously, radiographic screening is not employed routinely for individuals of average risk. Cross sectional imaging is typically prompted by reported symptoms warranting further investigation, wherein a mass may or may not be discovered. This poses a substantial obstacle to early identification of disease in PDAC where symptoms do not typically arise until disease has already reached an advanced stage, precluding curative-intent treatment with surgery. When a mass is identified within the pancreas, a major obstacle faced in obtaining a tissue biopsy involves the requirement for sophisticated endoscopic techniques by an interventional gastroenterologist, which are not widely available in more rural areas. Acquiring adequate tissue for pathologic diagnosis may additionally be infeasible or pose undue risk to the patient, such as if the mass is in a poorly accessible location or in the setting of acute illness. In this case, liquid biopsy provides a non-invasive alternative and potential surrogate to tissue biopsy in the diagnosis of PDAC, with the added benefits of affordability, rapidity of results, and low risk of harm to the patient from sample acquisition. Although there is strong evidence that liquid biopsy may be an equivalent surrogate for tumor tissue in PDAC with complete or near-complete concordance between mutations detected in ctDNA and tumor tissue, many studies have found less than 50% concordance in early stages of disease, and there continues to be speculation about its use as an equally strong or better alternative (6, 21, 22). A potential reason for variation in concordance between ctDNA and tumor tissue is both intratumoral and intertumoral heterogeneity (23). As ctDNA is collected peripherally and is not limited to subsection analysis as in the case of traditional tissue biopsy, it is favored to capture tumor heterogeneity more broadly and comprehensively (24, 25). There have been multiple studies supporting analysis of ctDNA and CTCs on top of tissue biopsy due to their discordance with tissue acquired from the primary tumor, as actionable biomarkers informative of tumor behavior have been found to be present in these specimens that were not detected in tissue samples (21, 26). Further improvements in sequencing technology for ctDNA analysis and standardization of sequencing platforms across clinical studies are needed to further evaluate ctDNA as an equivalent surrogate for tissue biopsy, however its ability to capture tumor heterogeneity for more comprehensive disease profiling is an undisputable unique benefit and favors its use at least in conjunction with tissue analysis.

## Circulating tumor DNA as a novel and independent biomarker for PDAC management

Despite the use of multiple imaging modalities and blood marker analysis, identification of resectable lesions is challenging and minimal increases in diagnosis of PDAC at resectable stages over the past two decades highlights the sufficient need for a novel, highly sensitive and disease-specific biomarker that can improve early and precise diagnosis for more patients (27). Herein lies the benefit of ctDNA analysis, with its potential as an independent patient-specific biomarker that may aid in accurate and prompt diagnosis of early-stage PDAC.

The only currently validated biologic marker in PDAC is cancer antigen 19-9 (CA 19-9), having been considered the gold standard biomarker for the diagnosis and monitoring of PDAC for over three decades (28). However, CA 19-9 is not an independent diagnostic marker and is usually used in addition to radiographic imaging in clinical practice, despite its overall sensitivity of 78.2% and 82.8% specificity for presence of PDAC (29). Multiple studies have correlated ctDNA abundance with CA 19-9 levels, with the added benefit of much fewer false positive rates and higher detectability for ctDNA fragment analysis (30, 31). In addition, analysis of ctDNA is much more specific than other blood-based biomarkers that are typically present at various levels as detection of any measurable amount of tumor DNA indicates the presence of malignancy (32). While studies provide evidence supporting ctDNA as a strong and even independent diagnostic marker, it is present in considerably smaller quantities in early disease which poses an obstacle to its use as an early diagnostic tool (33). However, its use alongside current biomarkers shows strong promise for improving precision in early diagnosis. A study by Cohen et al. in 2018 demonstrated that a combined assay combining both CA 19-9 and ctDNA abundance demonstrated superior precision over use of any biomarker alone as a diagnostic tool for early PDAC, demonstrating the ability to detect disease in 60% of patients who had no traditional presenting symptoms of PDAC as well as 41% of PDAC patients who would be diagnosed with Stage I disease (34). Another work by Majumder et al. in 2021 demonstrated that presence of specific methylated DNA markers (MDMs) in ctDNA outperformed CA 19-9 in accurately detecting PDAC cases, and that combined use of an MDM panel and serum CA 19-9 was superior to use of either alone, demonstrating a sensitivity of 92% and specificity of 97.5% (35). While study results support that ctDNA improves diagnostic accuracy alongside other biomarkers, including the current standard of care marker CA 19-9, further studies are needed to further evaluate ctDNA’s ability to outperform CA 19-9 as well as its strength as an independent biomarker for diagnostic use.

**Figure 1:**
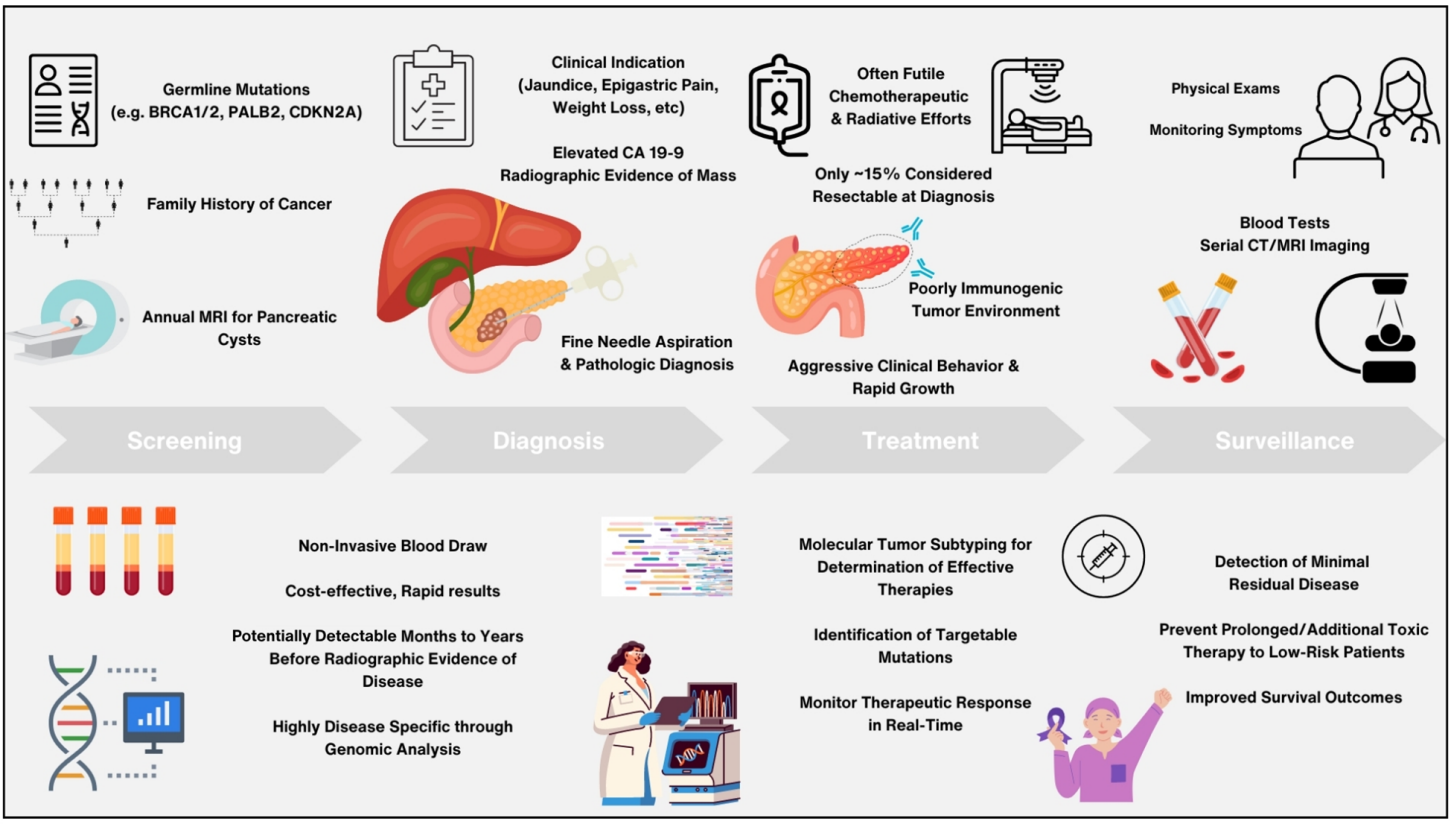
Schema detailing benefits and promise of circulating tumor DNA analysis versus current standard of care methods in the management of pancreatic adenocarcinoma throughout screening, diagnosis, treatment, and surveillance.

Beyond diagnosis, CA 19-9 is also used throughout the treatment course to assess response and disease clearance after curative-intent resection and/or therapy. Decreasing levels of CA 19-9 from baseline measurements have been shown to be associated with improved overall survival and indicative of positive treatment response, whereas increasing CA 19-9 levels while on active treatment have been associated with lower survival (36, 37). Similar results have been demonstrated by ctDNA analysis throughout the treatment course as well as pre- and post-operatively in many studies, with clearance of ctDNA after therapy and/or surgery being strongly prognostic of improved survival and supportive of a favorable response to treatment (38). In multiple studies evaluating localized PDAC, ctDNA abundance has also been shown to correlate with radiographic disease burden as well as the presence of distant metastases (39). As illustrated in **Figure 1**, ctDNA analysis thus demonstrates immense potential for improving PDAC management through prompt and accurate diagnosis, stratification of patients, and guidance of clinical decision making, providing an abundance of benefit beyond what current biomarkers offer (40).

## Enhancing patient stratification, personalized therapy, and surveillance with ctDNA

Contributing to the poor survival in PDAC is its notable resistance to chemotherapy, immunotherapy, and radiotherapy (41). With the limited genomic profile of PDAC involving just four key genes (KRAS, CDKN2A, SMAD4, TP53), the presence of KRAS mutations in over 90% of tumors is favored to drive the disease’s notoriously aggressive behavior (42). It is PDAC’s large dependency on the KRAS gene that makes it a popular target of interest in the liquid biopsy sphere in hopes of further elucidating this disease type to improve PDAC management. Multiple studies have identified acquisition of a KRAS mutation to be an early tumorigenic event responsible for contributing to the progression of high-grade dysplasia to PDAC, making KRAS an ideal target for analysis in early stages of disease (43). The presence of pertinent KRAS mutations – most commonly the G12D, G12V, and G12R variants which together represent over 90% of KRAS mutations in PDAC - harbored by ctDNA have been shown to be prognostic in early-stage disease (44). Studies detecting higher amounts of KRAS mutations in ctDNA, particularly the more aggressive G12D and G12V variants, have been associated with more aggressive and higher stage disease, presence of micrometastasis and liver metastases, and worse overall survival (45, 46). A study by Nitschke et al. in 2023 upheld these findings in a cohort including resectable and advanced PDAC, demonstrating that while fewer curative patients had high copy numbers of mutant KRAS ctDNA, it remained a strong and independent predictor of shorter relapse-free and overall survival (47). In another study by Shah et al. in 2024, baseline mutant KRAS ctDNA levels were independently predictive of worse overall survival even in patients who underwent neoadjuvant treatment, surgery, and adjuvant therapy (48). Vitello et al. in 2024 further evaluated pertinent KRAS mutations in ctDNA both pre- and post-operatively in resectable PDAC patients who received neoadjuvant therapy, demonstrating that clearance of KRAS ctDNA after treatment was significantly predictive of improved overall survival while detection of mutant KRAS in ctDNA after resection, particularly the KRAS G12V variant, was associated with worse overall survival (49).

Analysis of KRAS mutations in ctDNA has also shown benefit in the surveillance setting, providing benefit to patients beyond those on active treatment. Watanabe et al. in 2019 performed sequential analysis of ctDNA in 39 patients who underwent surgery for resectable disease, with findings demonstrating that emergence of mutant KRAS ctDNA was significantly prognostic regardless of disease recurrence while CA 19-9 levels were predictive of recurrence and not prognosis, and that non-emergence of mutant KRAS ctDNA within 1 year after surgery was predictive of better prognosis (50). Groot et al. in 2020 similarly reported that detectable mutant KRAS ctDNA during long term follow up post-pancreatectomy predicted disease recurrence, with demonstrated sensitivity of 90%, specificity of 88%, and overall diagnostic accuracy of 89% (51).

Additionally, the more aggressive molecular subtype of PDAC, termed basal-like, has also been shown to carry higher copy numbers of KRAS G12V mutations by Martinelli et al. in 2017, which may be suggestive of greater susceptibility to KRAS pathway targeting over cytotoxic agents (52). Analysis of non-KRAS genetic aberrations in ctDNA additionally inform microsatellite instability and tumor mutational burden, which clinically inform susceptibility of disease to immunotherapeutic agents (53). As disease is shown to progress clinically, alterations in KRAS mutations harbored in ctDNA as well as their abundance have been shown in a small pilot study to correlate with both radiologic imaging results and CA19-9 levels, demonstrating the ability of ctDNA monitoring on treatment to suggest early resistance or sensitivity to specific therapies or indicate early progression of disease, making serial analyses throughout treatment a promising tool for potentially guiding clinical decisions (54). While current data is limited, further studies evaluating the molecular profile through ctDNA to identify newly evolving tumor behavior a and potentially exploitative mutations for specific therapeutic agents or targeted treatments are necessary to improve PDAC management.

## Clinical trials investigating the role of ctDNA analysis in early PDAC detection and management

Evaluation of ctDNA in clinical trials to verify its role in predicting prognosis, determining treatment efficacy and guiding clinical decisions in early PDAC have been underway in recent years. One of the earliest observational studies in PDAC, the PANC-CTC Trial published results in 2019 demonstrating that liquid biopsy combining multiple biomarkers including circulating tumor cell showed superior results in sensitivity and negative predictive value to current combined diagnostic technologies (fine needle aspiration and serum CA 19-9 result) and proved a strong diagnostic tool for early, potentially curable PDAC (55). The ongoing observational CASPER Study aims to evaluate the prognostic value as well as predictive value for treatment response of ctDNA in patients with resectable PDAC undergoing either upfront or interval surgery following neoadjuvant treatment (56). The Phase I AMPLIFY-201 successfully evaluated the efficacy of a novel mutant-KRAS targeted cancer vaccine ELI-002 2P based on reduction or clearance of KRAS-mutant ctDNA in PDAC following resection and locoregional therapy completion, with the Phase I/II AMPLIFY-7P trial now underway to further evaluate if clearance of mutant-KRAS ctDNA in response to ELI-002 2P administration is predictive of improved relapse-free survival (57, 58). The PRIMUS-002 trial aims to evaluate response to neoadjuvant chemotherapy in resectable and borderline resectable PDAC based on mutations harbored in ctDNA indicating an intact or defective DNA damage response with aims to exploit this information for treatment selection (59). Early results from the prospective multi-center DYNAMIC Pancreas Trial published in May 2024 demonstrated the feasibility of using a ctDNA-guided approach to inform clinical decisions to administer adjuvant chemotherapy to patients who are ctDNA positive after upfront or interval resection (60). After results from the COMPASS Trial in 2020 demonstrated improved objective responses to matched chemotherapy based on molecular subtype determined from tumor biopsy results in metastatic PDAC, the ongoing observational ACCELERATE Study aims to evaluate if ctDNA results are similarly capable of accurately informing treatment decisions and optimal regimen selection in a PDAC population that includes resectable patients (61). The results of these major trials are further summarized in **Table 1**.

**Table 1:** Summary of clinical trials investigating the role of circulating tumor DNA as a biomarker in early-stage PDAC.

Clinical Trials Evaluating the Role of Circulating Tumor DNA and Liquid Biopsy in Early PDAC	Study Dates	Status	Study Goal	Findings
ACCELERATE Study (Using Tumour DNA and Proteins to Better Understand How Pancreatic Cancer Responds to Treatment)	November 2024 Estimated Study Completion in December 2031	Ongoing	To validate if liquid biopsy is feasible to obtain genetic test results within a timeframe that can help inform treatment decisions and if ctDNA analysis results can successfully predict treatment response in early-stage PDAC.	
AMPLIFY-201 Phase Study	October 2021 Estimated Study Completion in March 2026	Ongoing. Early Results	To evaluate efficacy of lymph-node targeted cancer vaccine ELI-002 2P in PDAC and colorectal cancer patients status-post surgery and locoregional therapy who are considered high-risk for recurrence based on alterations in ctDNA levels harboring G12D and G12R mutant KRAS	-ELI-002 2P was safe with demonstrated ctND and serum tumor biomarker reduction in 79% of patients and clearance in 21% of patients. as well as notable mutant KRAS-specific T cell immune responses in 8-% of patients Results suggest that evaluation of cDNA reduction/clearance in resectable PDAC patients who are cDNA-positive after curative intent treatment is an effective method to accurately determine improved RFS
AMPLIFY-7P Phase I/II Study	April 2023 Estimated Study Completion in November 2026	Ongoing	To evaluate the ability of ELI-002 2P to delay disease recurrence compared to observation in patients with RAS-mutated PDAC who have undergone surgery and completed curative intent locoregional treatment based on reduction or clearance of cIDNA. or if cDNA is undetectable serum tumor antigen CA 19-9	
CASPER Study (Circulating Tumor DNA as Surgical Biomarker in Patients With PanerFatic Adenocarcinoma for Statement of Resectability)	December 2022 Estimated Study Completion in May 2026	Ongoing	To evaluate the prognostic value of ctDNA as a marker of surgical futility. prognosticate patients. and/or predict the treatment response in patients with resectable PDAC	
DYNAMIC Pancreas, Phase II Non-Randomized Study	March 2019 November 2023	Ongoing Early Results	To evaluate the feasibility of post-operative CIDNA levels to improve risk stratification and/or provide real time indication of adjuvant chemotherapy benefit for resectable PDAC patients.	-Post-operatively, CA 19.9 was elevated in 29% of patients versus ctDNA was detectable in 40% of patients Post-operative ctDNA status was significantly predictive of recurrence-free survival, with ADNA positive patients demonstrating worse RFS compared to patients who were cDNA negative (13 months versus 22 months: HR 0.52. P - 0.003). A large proportion of patients with resectable PDAC have detectable ctDNA post-operatively and post-operative cDNA levels are independent of known prognostic markers (i.e., CA 19-9)- Post-operative measurement of ctND levels in resectable PDAC status-post resection may be an attractive way to monitor MRD predict RFS/OS and guide clinical decision making
PANC-CTC Study	February 2017 November 2017	Completed	To evaluate the combined diagnostic performance and prognostic value of liquid biopsy and circulating biomarkers (CTCs. KRAS mutant alleles and tumor-specific exosomes) to identify ideal candidates for upfront surgery in resectable PDAC patients using four different assay platforms.	-Combining CTC and GPC1-positive exosome detection displayed 100% of sensitivity and 80% of specificity with negative predictive value of 100% [95% C1j. as compared to combined conventional tools of serum CA 19-9 and EUS FNA which demonstrated 50% sensitivity and 92% specificity with a negative predictive value of 70% [95% CIJ.- Concomitant detection of several circulating tumor biomarkers (CTCs and tumor-specific exosomes) carry high diagnostic value and successfully identify patients at risk of early disease relapses and/or fatal outcomes. and is a desirable approach for PDAC diagnosis as it may greatly accelerate diagnosis and may help guide clinical decision making.
PRIMUS-002. Phase II Non-Randomized Study	April 2019 July 2021	Further Work Ongoing. Early Results	To evaluate the predictive value of intact or defective DNA damage response (DDR) pathways determined from liquid biopsy results using novel DDR assay to predict response to either of two neoadjuvant therapy regimens (FOLFOX-Abraxane versus Gemcitabine-Abraxane) in patients with resectable PDAC.	-Both regimens of FOLFOX-Abraxane and Gemcitabine-Abraxane administered neoadjuvantly to resectable PDAC patients are effective and safe, with median survival of 23.7 months (12.9 months to 24.9 months, 90% confidence interval) for the FOLFOX-Abraxane arm. and 20.5 months (8.9 months to NA: 90% confidence interval) for the Gemneitabine-Abraxans arm. Further work is underway to evaluate if DDR deficiency as determined by molecular profiling of ctDNA is predictive of selective sensitivity to a platinum-based (FOLFOX) regimen

As ctDNA analysis continues to show promising results in clinical trials, there is significant momentum carrying it closer to routine clinical practice. Further studies are needed to solidify the precise clinical role of ctDNA in PDAC, whether it be as a companion diagnostic, for tumor subtyping and regimen selection, or as a monitor of disease recurrence in the adjuvant or surveillance setting.

### Methods for ctDNA analysis in the clinical setting

Currently, the high-throughput PCR-based method of Next-Generation sequencing (NGS) is the most promising and widely used method for evaluating tumor-specific mutations in ctDNA via liquid biopsy (62). The use of NGS-based methods provides extensive genome-wide information, as it allows for detection of multiple tumor-associated genetic and epigenetic aberrations found in a patient’s serum ctDNA by analyzing findings against a healthy reference. Employing NGS methods is therefore highly effective for establishing a comprehensive tumor profile of mutated variants (63). However, it has drawbacks in clinical use, being costly and time inefficient, while additionally requiring complex interpretation. These aspects make it difficult to employ for the monitoring of response to therapy over time or in the surveillance setting. However, development of optimized NGS panels limiting analysis to the most commonly mutated genes in PDAC have shown strong results in a recent study, providing a potential solution for the drawbacks currently facing large NGS panels analyzing hundreds of cancer-associated genes without sacrificing accuracy.

Validation of specific mutant DNA sequences detected via NGS through digital droplet PCR (ddPCR) allows for improved detection of rare mutations with variant allele frequencies of <0.01%, which are common in PDAC given its low ctDNA shed rate (62–64). Furthermore, ddPCR has the added ability to effectively analyze incredibly small quantities of ctDNA, being vital in the case analyzing pertinent mutations in early stage PDAC were ctDNA levels can be extremely low, with study results demonstrating the ability to detect mutant KRAS in ctDNA even in non-neoplastic pancreatic disease (65). Single-cell analysis also provides targeted detection at a lower cost, but in turn lacks the broad mutational assessment provided by NGS or the high sensitivity of ddPCR, making them less favorable. For example, single-cell methods allow for a detailed examination of isolated CTCs, capturing cellular heterogeneity and rare mutations or distinct subpopulations that bulk cell analysis may overlook. Isolated single cell analysis provides the resolution to analyze individual cell characteristics that may reveal unique genomic and transcriptomic profiles that can provide deeper insight on tumor evolution and behavior (66). However, while this focused approach may offer deeper insights into cancer cell evolution and resistance mechanisms, it is more expensive, technically complex, and less established for clinical use than NGS or ddPCR. Furthermore, single-cell techniques lack the ability to broadly analyze tumor heterogeneity, being limited to analyzing the properties of a single cancer cell.

Beyond genomic sequencing via PCR-based methods, the rise of multi-omics technologies in recent years has provided the opportunity to go beyond the genome and obtain transcriptomic and proteomic data for phenotyping of tumors. Multi-omics also provides information regarding tumor behavior beyond the genome for personalized treatment curation (67). For example, transcriptomic and proteomic signatures in CTC’s have been evaluated against tumor mutational profiles to identify resistance to certain therapeutic agents and thus allow for optimal treatment curation individualized to the molecular signature of each patient (68).

The evaluation of epigenetic modifications in ctDNA additionally provides the opportunity to obtain information not reflected in genomic analysis, as tumor behavior and expression of specific genes involved in key molecular pathways can be further evaluated. This information is important, as many genomic abnormalities may not manifest transcriptionally or phenotypically, making the sole use of genomic analysis of ctDNA suboptimal for comprehensive disease profiling as it may be misleading. Methylation, acetylation, and other epigenetic modifications may also reflect tumor molecular subtypes, therefore being important for informing prognosis and therapeutic selection (69). Earlier studies have validated genome methylation profiles that accurately distinguish healthy individuals from those with cancer. Recent studies have shown ctDNA methylation to be identical to that of the primary tumor as well as highly correlative with common oncogenic signaling pathways, frequency of mutations and susceptibility to therapies such as those targeting PD-1 and PDL-1 (70, 71).

These findings highlight the ability of a pan-omics approach in ctDNA analysis to gain more in-depth information about tumor behavior and characteristics than ever, thus further personalizing medicine and oncologic care. Employment of any method depends on the specific requirements and goals of the study or clinical scenario, considering the cost and scope of genetic analysis needed. Given the lack of consensus on which technique for ctDNA assessment is optimal, a combined or pragmatic approach is likely to be most useful. The method of ctDNA assessment or assessments should, therefore, be selected with regard to the advantages and disadvantages of the various techniques, available technology, and familiarity with assessing results. Further prospective studies will be needed to definitively establish the clinical utility of techniques and determine the most effective methodology for potential application into routine clinical practice.

## Challenges and future directions of ctDNA in early PDAC care

Circulating tumor DNA analysis in PDAC shows significant promise for improved early detection and disease management. However, its application in clinical practice is hindered by several current challenges. First, the lack of consensus on the method of assessment presents a major hurdle in developing an effective and affordable methodology by supporting consensus for widespread use. Many studies use different methods of assessment with no one technique emerging superior. Further studies comparing performance between techniques are therefore needed. Moreover, ctDNA analysis is presently lacking evidence to be used independently, requiring concomitant use with other prognostic markers, imaging studies, and tissue biopsy. This interdependence complicates clinical workflows, as healthcare providers would need to integrate multiple diagnostic modalities to arrive at definitive assessments with the additional drawback of increasing the complexity and cost of care for patients. The reliance on these traditional methods emphasizes that ctDNA, while promising, is not currently a standalone solution but rather a complementary tool that requires careful interpretation in the context of comprehensive clinical evaluation. However, as ctDNA is non-invasively obtained and provides unique benefits in evaluating treatment response and tumor subtyping, its use as an adjunct is supported by its broad benefits compared to minimal added costs.

Another significant issue is the biological variability inherent in PDAC tumors. Many patients may not shed detectable levels of ctDNA in addition to PDAC lacking a strong molecular profile outside of *KRAS* aberrations, which happens to be the most mutated oncogene in all human malignancies. With minimal amounts of tumor shedding in PDAC along with a limited molecular profile, ctDNA analysis may serve little to no benefit for individuals with nonspecific mutations or undetectable ctDNA. Additionally, with the majority of PDAC patients not having surgically resectable disease at diagnosis, it is unclear how disease properties and thus ctDNA are altered as disease progresses (72). With many recent works identifying major differences in tumor behavior, metabolism, and molecular character between localized versus metastatic disease, the importance of ctDNA analysis is furthered given its ability to discriminate between the two (33, 34, 73). Furthermore, the contemporary emphasis on monitoring *KRAS* mutations in ctDNA, while crucial in PDAC due to their presence in over 90% of cases, highlights the broader issue of relying on a single biomarker. This may limit understanding of disease dynamics as well as restrict the benefit provided that ctDNA analysis may offer if restricted to single genes. Efforts to establish large genomic data sharing platforms from patients will help improve the current lack of knowledge on molecular profiles of human malignancies as well as to help identify strong target genes to focus analysis for streamlined use of ctDNA assay.

Aside from fundamental barriers, gaps in study design and evaluation warrant attention. One pressing issue is the limited evaluation of ctDNA in certain populations. Understanding the differences in ctDNA across disease stages is critical for refining ctDNA’s role as a biomarker for all patients, and further large studies are needed. Additionally, patients with concurrent malignancies or germline mutations are also often excluded from ctDNA studies due to expected discrepancies in mutation detection, which may lead to skewed understanding of ctDNA’s relevance in these patients. The dynamics of ctDNA shedding and profiling in these populations remain largely unexplored, and lack of evaluation in these cohorts limits the ability to generalize findings across diverse patient populations, as well as hampers our understanding of how genetic predispositions and presence of multiple cancer profiles may influence the dynamics of ctDNA and its analysis.

Long-term follow-up studies using ctDNA are additionally scarce, particularly as it remains a relatively novel technique on top of recent major gains in our understanding of the human genome due to rapid technological advancements in the past two decades. Without extended serial ctDNA assessments with longer periods of patient surveillance, our understanding of how ctDNA levels correlate with treatment outcomes or disease recurrence and progression in the long-term is lacking. As with any novel finding, it will take time to establish ctDNA’s role as an accurate tool for monitoring disease recurrence and in assessing treatment efficacy before there is strong enough data to support its movement into clinical practice.

The need for diversity in study populations is another critical gap. Many existing studies lack representation from ethnically, racially, and geographically diverse populations, which raises concerns about the applicability of findings to broader patient groups. To ensure that ctDNA analysis is a reliable tool for all patients, it is essential to evaluate its effectiveness across diverse demographics. Accessibility is a further barrier that must be addressed in order to bring the benefit of this novel methodology to all patients. While ctDNA analysis shows promise, it must be emphasized in further works that optimal analysis methods be widely available and affordable to all patient populations to ensure equitable healthcare access. This is particularly important for underserved communities who may not have the resources to benefit from innovative diagnostic tools and who often face more aggressive disease. With PDAC demonstrating continued rise in incidence with minimal gains in survival despite efforts in disease management, it is vital that support for work on novel advancements continue to improve outcomes for patients.

Despite current obstacles, the future of ctDNA analysis remains promising. Early detection of pancreatic adenocarcinoma is crucial for improving survival rates, particularly given the aggressive nature of the disease. With advancements in ctDNA analysis there is potential to identify the disease at its earlier stages when curative surgical interventions may be possible. Early detection would significantly alter patient outcomes, transforming pancreatic cancer from a typically lethal diagnosis to a much more manageable condition. Moreover, ctDNA can play a pivotal role in monitoring MRD in patients who undergo curative surgery or who have reached remission after adjuvant chemotherapy to spare those who are at low risk for recurrence from further toxic therapy for minimal benefit (74, 75).

The additional promise of personalized treatment curation using ctDNA is another significant, upcoming development. If optimized for clinical use, rapid results on serial samples in the clinic could be used by clinicians to guide regimen selection and to make informed treatment decisions for each patient. The promise provided by molecular analysis of PDAC through ctDNA allows for the gain of critical information about a relatively elusive cancer type, allowing for more tailored approaches to treatment, enhancing efficacy and minimizing adverse effects. With early and real-time monitoring of response to chemotherapy as well as identification of any evolving resistance mechanisms throughout the treatment course, longitudinal monitoring throughout treatment could serve to guide prompt clinical decisions and lead to better disease control. This is paramount in PDAC where therapeutic response is highly variable from patient to patient, and where patients may benefit from real-time monitoring throughout treatment. This paradigm shift offers hope for the future of PDAC management, as subtyping enables more accurate assessment of tumor biology with the potential to individualize treatment efforts to each patient.

## Conclusions

While challenges remain in the field of ctDNA analysis for pancreatic adenocarcinoma, the potential for its advancement and use in clinical practice is vast. Continued evaluation of its potential and validity in large trials will be paramount for moving the benefits provided by liquid biopsy and circulating tumor DNA analysis to patients, paving the way for early diagnosis and more effective treatment strategies to ultimately improve patient outcomes for such an aggressive disease.
